# Tobacco and E-Product Use by US Adults With Disabilities

**DOI:** 10.1001/jamanetworkopen.2024.60471

**Published:** 2025-02-18

**Authors:** Michael J. Parks, Daniela Marshall, Heather L. Kimmel, John H. Kingsbury, Colm D. Everard, Eiman Aboaziza, Carlos Blanco, Wilson M. Compton

**Affiliations:** 1 National Institute on Drug Abuse, National Institutes of Health, Bethesda, Maryland; 2 Kelly Government Solutions, Rockville, Maryland; 3 Axle Informatics, Rockville, Maryland

## Abstract

**Importance:**

Research on cumulative disabilities and tobacco use in the current tobacco landscape is lacking.

**Objective:**

To examine prevalence of tobacco use across cumulative disabilities within and across years among US adults.

**Design, Setting, and Participants:**

Representative cross-sectional surveys of the US adult (aged ≥18 years) civilian, noninstitutionalized population from the Population Assessment of Tobacco and Health (PATH) Study were compared across 2013 to 2014 (wave 1), 2016 to 2018 (wave 4), and 2022 to 2023 (wave 7). Age-adjusted estimates and change scores were compared using design-based significance tests. Data were analyzed from November 2023 and April 2024.

**Main Outcomes and Measures:**

Past 30-day use of tobacco (cigarette, electronic nicotine [e-product], traditional cigar, filtered cigar, cigarillo, pipe, hookah, and smokeless products); disability types (mobility, self-care, independent living, cognitive, hearing, and vision) summed to generate a 4-level cumulative disability measure (0, 1, 2, or ≥3).

**Results:**

In 2013 to 2014, among 32 314 participants at wave 1, 3110 participants were 65 years or older (18.2%), 15 993 (51.9%) were female, 5536 (15.2%) were Hispanic, 4496 (11.2%) were non-Hispanic Black, 19 295 (66.0%) were non-Hispanic White, and 2428 (7.5%) were another non-Hispanic race. A total of 32 314 adults were included in 2013 to 2014, 33 638 in 2016 to 2018, and 30 681 in 2022 to 2023. In 2013 to 2014, any tobacco use prevalence for those with no disabilities was 25.4% (95% CI, 24.7% to 26.1%), while prevalence was 40.2% (95% CI, 38.6% to 41.8%), 48.9% (95% CI, 45.4% to 52.5%), and 51.8% (95% CI, 48.6% to 55.0%) for those with 1, 2, and 3 or more disabilities, respectively. Any tobacco, cigarette, e-product, cigarillo, filtered cigar, and pipe use were consistently higher among those with multiple disabilities compared with those with 0 or 1 disability. Prevalence was highest among those with 3 or more disabilities for all products. Tobacco use declined over time, except e-product use increased. However, not all declines in prevalence between 2013 to 2014 and 2022 to 2023 were significant among those with 3 or more disabilities. The declines for those who used any tobacco (−4.6%; 95% CI, −8.7% to −0.6%) and cigarettes (−8.9%; 95% CI, −12.7% to −5.1%) were small, and increases in e-product use were large (4.6%; 95% CI, 1.0% to 8.3%) for those with 3 or more disabilities compared with other groups.

**Conclusions and Relevance:**

In this survey study, disparities in tobacco use existed across cumulative disabilities. Despite declines in tobacco use over time, differences in use across cumulative disabilities persisted or worsened, especially among those with 3 or more disabilities. Targeted prevention, screening, and cessation efforts inclusive of multiple products are needed.

## Key Points

**Question:**

Does tobacco use prevalence vary among adults with 1 vs more disabilities, and has it changed across time?

**Findings:**

In this survey study of 32 314 participants at wave 1, cumulative disabilities were positively associated with tobacco use; prevalence was consistently highest for those with 3 or more disabilities, regardless of tobacco product type. Differences were stable over time or increased, despite overall declines in tobacco use prevalence.

**Meaning:**

These findings suggest individuals with disabilities were more likely to use tobacco, and this suggests they are more likely to experience health risks associated with tobacco use than those without disabilities.

## Teaser

This survey study evaluates the prevalence of tobacco use across cumulative disabilities within and across years among US adults.

## Introduction

Despite marked declines in combustible tobacco use (eg, cigarettes), tobacco use remains the leading preventable cause of mortality and morbidity in the US.^1,2,3,4^ Major disparities (associated with socioeconomic status, gender identity, sex, urbanicity, and mental health, among others) remain in terms of who uses these products.^4,5,6,7,8^ Additionally, new products such as electronic nicotine products (e-products) have emerged, and e-product use has increased in recent years (herein, e-product and tobacco are used interchangeably), especially among youths and young adults; and despite lower overall use rates, e-product use has also increased among older adults.^9,10,11^

Prevalence of tobacco use is higher among those who have severe mental or physical health conditions, or those who experience comorbidity (eg, multiple health conditions).^5,6,7,8,12^ More recent tobacco-related research has focused on individuals with disabilities (IWD). Approximately 1 in 4 adults in the US lives with a disability,^13^ demonstrating the public health importance of understanding disparities associated with disability status. Yet, IWD remain understudied in tobacco control research,^14,15,16,17,18^ even though IWD tend to use tobacco at higher rates than those without a disability.^15,16,17,18,19,20,21,22,23^

A limited amount of research has shown that some disability types are associated with use of certain tobacco products, while other disability types are not.^17^ However, gaps in research exist due to different analytic approaches and measures used to assess disability types, in addition to limited availability of generalizable data gathered at regular intervals, constraining our ability to document how IWD use a range of tobacco products relative to those without disabilities. More specifically, previous research has tended to examine individual disabilities rather than cumulative disabilities (ie, total number of disabilities),^24^ which is an important gap since this potential form of comorbidity may be a key tobacco-related disparity in the new tobacco landscape. Previous research has also not used nationally representative data gathered at frequent intervals (a strength of the Population Assessment of Tobacco and Health [PATH] Study), particularly over the period when tobacco use has declined overall. Finally, previous research has tended to assess a limited number of tobacco products due to small sample sizes and the rapidity with which use of different tobacco products emerge.^17^

Using data from 3 PATH Study cohorts, we examine prevalence of different tobacco product use across cumulative disabilities. First, we assess within-wave differences in prevalence across disabilities. Second, we examine across-wave differences in prevalence within groups based on number of disabilities. For all analyses, we assess the descriptive associations between cumulative disabilities and tobacco use, focusing on prevalence of use. Following research on comorbidity,^7,12^ we hypothesized that individuals who had more disabilities would have higher prevalence of tobacco use, regardless of product type. Mirroring trends in some other forms of comorbidity,^12,25,26,27^ we also hypothesized that the declines in tobacco use would be lower among those with the highest number of disabilities compared with individuals with fewer or no disabilities.

## Methods

The PATH Study is an ongoing, nationally representative, longitudinal cohort study of adults and youths in the US. The overall PATH Study sample consists of individuals selected at an inception wave and 2 later replenishment waves. At wave 1 (W1; data collected September 12, 2013, to December 14, 2014), the PATH Study employed a stratified address-based, area-probability sampling design that oversampled adults who use tobacco, young adults, and African American adults. Study participants recruited at W1 formed the W1 cohort. Further details regarding the PATH Study are published elsewhere.^28,29,30,31^ The study was conducted by Westat and approved by the Westat institutional review board. The study followed American Association for Public Opinion Research (AAPOR) reporting guidelines. All respondents aged 18 and older provided informed consent.

At wave 4 (W4), the first probability replenishment sample was selected from the US civilian noninstitutionalized population (CNP) at the time of W4 (data collected from December 1, 2016, to January 3, 2018). Details on PATH Study replenishment methods have been published elsewhere.^32^ W1 cohort individuals were combined with the W4 replenishment sample to form the W4 cohort. At wave 7 (W7), a second probability replenishment sample was selected from the US CNP at the time of W7 (data collected from January 6, 2022, to April 2, 2023). W4 cohort individuals were combined with the W7 replenishment sample to form the W7 cohort.

We used restricted use file (RUF) data from each cohort at their respective recruitment wave (W1 cohort at W1, W4 cohort at W4, and W7 cohort at W7), and assessed adults aged 18 years and older and youths who aged up into the adult study. There were 32 320 W1 cohort respondents at W1 (weighted response rate was 74.0%); 33 822 W4 cohort respondents at W4 (weighted response rates: 73.5% for W1 continuing sample and 68.0% for W4 replenishment); and 29 968 W7 cohort respondents at W7 (weighted response rates: 66.9% for W4 continuing sample and 55.1% for W7 replenishment). The study used audio computer-assisted self-interviews available in English and Spanish; however, W6 and W7 data collection could be administered either by telephone or in-person, and W7 data collection could be administered by telephone, in-person, or the web. Details on procedures, questionnaires, sampling, weighting, and response rates are described in the PATH Study RUF User Guide.^33^

### Measures

Current tobacco use, defined as past 30-day use, was measured by assessing separate dichotomous measures for 8 different tobacco product types (1 = yes, 0 = no), and 1 dichotomous measure that grouped separate tobacco products together to assess any tobacco use (1 = any product use, 0 = no use). Products assessed were combustible cigarettes, electronic nicotine products (eg, e-cigarettes), traditional cigars, filtered cigars, cigarillos, pipes, hookah, and smokeless (eg, chewing tobacco and snus).

Following previous research,^13,17^ we used 6 separate measures of disability types to assess whether individuals experienced limited functioning and participation in different domains and activities: mobility (ie, ambulatory, such as walking or climbing stairs), independent living (eg, running errands), self-care (eg, bathing), hearing, vision, and cognitive (eg, difficulty concentrating, remembering, or making decisions). We generated 6 dichotomous (1 = yes, 0 = no) measures (not mutually exclusive) to assess each respective disability. We generated a measure for cumulative disabilities^24^ by summing 6 disability types and generating a 4-level measure: 0, 1, 2, or 3 or more disabilities. Disability survey items were included in the initial survey only for the respective cohorts (W1 for W1 cohort, W4 for replenishment sample, and W7 for replenishment sample). Items were only assessed at W1 for the W1 cohort, and then only at each respective first wave for the W4 and W7 cohorts, and aged-up youths were only asked items at their first adult survey wave. Consequently, disability measures were based on responses at W1 through W3 for the W1 cohort, W4 through W6 for the W4 cohort replenishment sample, and W7 for the W7 cohort replenishment sample.

A 6-category ordinal measure (18-24, 25-34, 35-44, 45-54, 55-64, or ≥65 years) assessed age; a dichotomous measure assessed birth-assigned sex (1 = male, 0 = female). Race and ethnicity was a 4-category self-report measure: Hispanic, non-Hispanic Black, non-Hispanic White, and other non-Hispanic race and ethnicity (including Asian, multiple races, and other), and it was assessed to demonstrate the demographic breakdown of the PATH Study participants over time. Education was captured via a 4-category measure: less than high school, high school diploma, some college, and a college degree or more. Any missing data on age, sex, race, Hispanic ethnicity, and education were imputed as described in the PATH Study RUF User Guide.^33^ Imputation was used for less than 1% for each demographic measure at each wave, except race and ethnicity, which had less than 3% missing.

### Statistical Analysis

Prevalence estimates were generated for within-wave differences across number of disabilities, and design-based *F* tests determined statistical significance. For assessing prevalence change across waves (within groups), change scores were generated; 95% CIs determined if prevalence change was different from 0 (a corresponding statistical test and *P* value were also used). Following previous PATH Study methods,^34^ 2 change scores were generated for each estimate: prevalence change between (1) 2013 to 2014 (W1) and 2022 to 2023 (W7), and (2) between 2016 to 2018 (W4) and 2022 to 2023 (W7). Since disability status and age are correlated,^13^ age-adjusted estimates were generated for all analyses of tobacco use prevalence across disability measures. Full-sample and replicate weights (balanced repeated replication with the Fay adjustment set to 0.3) accounted for complex sample design, nonresponse, and allowed for repeated, cross-sectional comparisons across waves (including accounting for potential effects of dependence due to repeated measurements from the same individuals).^28,34^ The weights are constructed to ensure estimates represent the CNP adult population in the US at the time of each wave. Pairwise deletion was used for missing data on tobacco use and disability (missing data was <4% in all analyses). Data analyses were conducted with Stata version 16 (StataCorp) using the svy extension. Statistical significance was prespecified at *P* < .05 with 2-tailed tests and sensitivity analyses adjusted for multiple comparisons. Data were analyzed between November 2023 and April 2024. Supplemental analyses assessed differences in tobacco use within waves for individual disabilities and any disability (1 = any disability, 0 = no disabilities), and weighted logistic regressions that included tobacco use measures as outcomes were used to test whether the interaction between survey year (wave) and cumulative disabilities were statistically significant (ie, to determine if changes in prevalence over time varied by disability status; see eMethods in Supplement 1).

## Results

### Descriptive Results

A total of 32 314 adults were included in 2013 to 2014 (W1), 33 638 in 2016 to 2018 (W4), and 30 681 in 2022 to 2023 (W7). In 2013 to 2014, 3110 participants were 65 years or older (18.2%), 15 993 (51.9%) were female, 5536 (15.2%) were Hispanic, 4496 (11.2%) were non-Hispanic Black, 19 295 (66.0%) were non-Hispanic White, and 2428 (7.5%) were other non-Hispanic. There were significant changes in prevalence of demographic groups over time, and all demographic information across 2013 to 2014, 2016 to 2018, and 2022 to 2023 is listed in Table 1.

**Table 1.** Weighted Age-Adjusted Prevalence of Tobacco Product Use Across Number of Disabilities and 3 Waves of Population Assessment of Tobacco and Health Study

Characteristic	Wave 1 (September 2013 to December 2014)		Wave 4 (December 2016 to January 2018)		Wave 7 (January 2022 to April 2023)	
	Participants, No./Total No.^a^	Weighted % (95% CI)	Participants, No./Total No.^a^	Weighted % (95% CI)	Participants, No./Total No.^a^	Weighted % (95% CI)
Tobacco use						
Any tobacco product	17 783/31 646	29.6 (28.9-30.3)	15 685/33 642	27.6 (27.0-28.3)	10 592/29 780	24.5 (23.8-25.2)
Cigarettes	14 219/32 284	22.5 (21.9-23.2)	11 989/33 638	21.2 (20.6-21.7)	6341/29 772	15.6 (15.1-16.1)
E-products	4430/32 246	6.7 (6.4-7.0)	4050/33 611	6.2 (6.0-6.5)	4741/29 765	8.9 (8.6-9.3)
Cigar	2137/31 959	3.6 (3.4-3.7)	1868/33 623	3.4 (3.2-3.6)	1062/29 769	2.7 (2.5-2.9)
Cigarillo	3060/31 303	4.4 (4.2-4.7)	2942/33 613	4.3 (4.1-4.5)	1374/29 766	2.8 (2.6-3.0)
Filtered cigar	1185/31 309	1.8 (1.7-2.0)	1166/33 616	1.8 (1.7-2.0)	485/29 765	1.1 (1.0-1.2)
Pipe	583/32 248	0.9 (0.8-1.0)	469/33 630	0.8 (0.7-0.9)	222/29 773	0.5 (0.5-0.6)
Hookah	1719/32 261	2.2 (2.0-2.4)	1242/33 629	1.8 (1.7-1.9)	661/29 769	1.3 (1.2-1.4)
Smokeless	1825/32 001	3.0 (2.8-3.2)	1585/33 629	3.0 (2.8-3.2)	676/29 767	2.1 (1.9-2.3)
Disabilities						
Number of disabilities						
0	23 621/32 286	75.2 (74.4-76.0)	24 918/33 621	76.0 (75.2-76.8)	22 303/29 763	77.4 (76.5-78.2)
1	5076/32 286	14.5 (14.0-15.0)	5279/33 621	14.6 (14.1-15.2)	4650/29 763	14.5 (13.9-15.0)
2	1949/32 286	5.4 (5.1-5.8)	1976/33 621	5.2 (4.9-5.5)	1737/29 763	4.9 (4.6-5.3)
≥3	1640/32 286	4.9 (4.5-5.3)	1448/33 621	4.2 (3.9-4.6)	1073/29 763	3.3 (2.9-3.6)
Type of disability						
Any disability	8665/32 286	24.8 (24.0-25.6)	8703/33 621	24.0 (23.2-24.8)	7460/29 763	22.7 (21.8-23.5)
Mobility	4265/32 224	13.3 (12.7-13.9)	3859/33 566	12.3 (11.8-12.9)	2630/29 726	10.0 (9.5-10.7)
Independent living	2155/32 233	6.1 (5.8-6.5)	2170/33 568	5.5 (5.1-5.8)	1929/29 712	4.9 (4.5-5.3)
Self-care	1072/32 250	3.3 (3.1-3.7)	904/33 588	2.8 (2.5-3.1)	669/29 744	2.3 (2.0-2.5)
Hearing	1424/32 255	5.3 (5.0-5.8)	1254/33 538	4.8 (4.5-5.2)	917/29 742	4.1 (3.7-4.5)
Vision	2123/32 253	5.5 (5.2-5.9)	2006/32 253	5.0 (4.6-5.4)	1588/29 739	4.6 (4.2-5.0)
Cognitive	3877/32 211	9.6 (9.1-10.1)	4215/33 538	9.7 (9.3-10.2)	4190/29 702	10.1 (9.6-10.7)
Demographics						
Age, y						
18-24	9109/32 303	13.0 (13.0-13.0)	11 213/33 638	12.4 (12.4-12.4)	10 310/29 778	11.7 (11.7-11.7)
25-34	6337/32 303	17.7 (17.3-18.2)	6874/33 638	17.9 (17.3-18.4)	6948/29 778	17.3 (17.0-17.7)
35-44	4930/32 303	16.5 (16.1-17.0)	4460/33 638	16.2 (15.6-16.7)	3548/29 778	16.9 (16.5-17.3)
45-54	4846/32 303	17.9 (17.5-18.4)	4142/33 638	16.7 (16.2-17.2)	2848/29 778	15.7 (15.1-16.3)
55-64	3971/32 303	16.6 (16.2-17.0)	3842/33 638	17.4 (16.8-17.9)	2984/29 778	16.8 (16.2-17.5)
≥65	3110/32 303	18.2 (18.2-18.3)	3107/33 638	19.6 (19.5-19.6)	3140/29 778	21.6 (21.6-21.6)
Sex						
Female	15 993/32 314	51.9 (51.9-52.0)	17 125/33 642	51.9 (51.9-51.9)	15 674/29 736	51.5 (51.5-51.6)
Male	16 321/32 314	48.1 (48.1-48.1)	16 517/33 642	48.1 (48.1-48.1)	14 062/29 736	48.5 (48.4-48.5)
Race and ethnicity						
Hispanic	5536/31 755	15.2 (15.2-15.3)	5152/33 642	15.7 (15.7-15.7)	2454/29 198	17.2 (17.1-17.2)
Non-Hispanic Black	4496/31 755	11.2 (11.1-11.3)	6672/33 642	11.7 (11.7-11.7)	4586/29 198	11.0 (10.9-11.1)
Non-Hispanic White	19 295/31 755	66.0 (65.9-66.2)	19 137/33 642	64.3 (64.3-64.3)	15 403/29 198	61.4 (61.3-61.5)
Other non-Hispanic^b^	2428/31 755	7.5 (7.4-7.6)	2681/33 642	8.3 (8.3-8.3)	6755/29 198	10.5 (10.3-10.6)
Education						
Less than high school	6488/32 314	16.7 (16.6-16.8)	6383/33 642	16.0 (15.9-16.0)	4468/29 780	14.1 (14.1,14.1)
High school diploma	7601/32 314	24.4 (24.3-24.5)	8161/33 642	23.8 (23.8-23.8)	7323/29 780	23.4 (23.4-23.4)
Some college	11 372/32 314	31.1 (30.9-31.2)	11 929/33 642	31.0 (31.0-31.0)	10 333/29 780	29.7 (29.7-29.7)
College degree or more	6853/32 314	27.8 (27.7-28.0)	7169/33 642	29.3 (29.2-29.3)	7656/29 780	32.9 (32.9-32.9)

^a^ Represents unweighted sample size for the numerator for each corresponding estimate with total unweighted number as the denominator.

^b^ Included Asian, multiple races, and other.

As shown in Table 1, prevalence of any product use was 29.6% (95% CI, 28.9% to 30.3%). Cigarette use was most prevalent in 2013 to 2014 during W1 (22.5%; 95% CI, 21.9% to 23.2%), followed by e-products (6.7%, 95% CI, 6.4% to 7.0%); prevalence of all other products was less than 5%. All product use declined over time except e-product use, which increased 2.3 percentage points by 2022 to 2023 (W7). In 2013 to 2014, 75.2% (95% CI, 74.4%-76.0%) had no disabilities; 14.5% (95% CI, 14.0%-15.0%), 5.4% (95% CI, 5.1%-5.8%), 4.9% (95% CI, 4.5%-5.3%) had 1, 2, and 3 or more disabilities, respectively. Disability prevalence remained stable over time; however, prevalence of no disabilities slightly increased over time (2.2 percentage points by 2022 to 2023), while prevalence of 3 or more disabilities declined (−1.6 percentage points by 2022 to 2023). Mobility disability was most prevalent in 2013 to 2014 (13.3%; 95% CI, 12.7% to 13.9%), followed by cognitive (9.6%; 95% CI, 9.1% to 10.1%), independent living (6.1%; 95% CI, 5.8% to 6.5%), vision (5.5%; 95% CI, 5.2% to 5.9%), hearing (5.3%; 95% CI, 5.0% to 5.8%), and self-care (3.3%; 95% CI, 3.1% to 3.7%) disabilities. Prevalence of any disability was 24.8% (95% CI, 24.0% to 25.6%). More disabilities were present among those with independent living and self-care disabilities in 2013 to 2014, while IWD with a hearing disability had the highest prevalence of a single disability (Figure); these patterns remained identical over time. Separate disabilities remained stable over time with minor declines in all except for cognitive disability.

**Figure.**
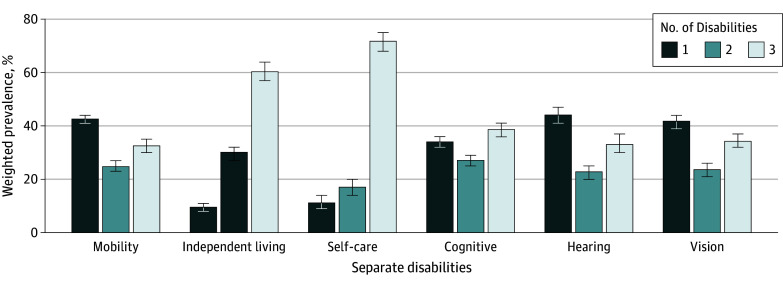
Weighted Prevalence of Number of Disabilities Individuals Experience Across 6 Separate Categories of Disability Types at Population Assessment of Tobacco Health Study Wave 1, 2013 to 2014

### Within-Wave Differences in Tobacco Use Across Cumulative Disabilities

In 2013 to 2014 (W1), prevalences of any tobacco, cigarette, e-product, cigarillo, filtered cigar, and pipe use were significantly higher among those with 1 or multiple disabilities compared with those with no disability (eTable 1 in Supplement 1). Prevalence in nearly all products increased with number of disabilities, and prevalence was consistently highest among those with 3 or more. For example, in 2013 to 2014, prevalence of any tobacco use for those with no disabilities was 25.4% (95% CI, 24.7% to 26.1%), while prevalence was 40.2% (95% CI, 38.6% to 41.8%), 48.9% (95% CI, 45.4% to 52.5%), and 51.8% (95% CI, 48.6% to 55.0%) for those with 1, 2, and 3 or more disabilities, respectively. Differences in cigar, hookah, and smokeless tobacco were minimal or nonsignificant (eTable 1 in Supplement 1). For example, prevalence of smokeless tobacco use was 2.8% (95% CI, 2.6% to 3.0%) for those with no disabilities, and 3.6% (95% CI, 3.1% to 4.1%), 3.5% (95% CI, 2.8% to 4.3%), and 3.9% (95% CI, 3.0% to 5.0%) for those with 1, 2, and 3 or more disabilities, respectively. Magnitude of differences were most marked for those with 3 or more disabilities compared with no disabilities: prevalence of any tobacco, cigarette, e-product, cigarillo, filtered cigar, and pipe use were, respectively, 2.04, 2.40, 2.65, 3.05, 4.20, and 4.57 times higher for those with 3 or more disabilities compared with those with no disabilities at W1.

Generally, all patterns in tobacco use were similar within 2013 to 2014 (W1), 2016 to 2018 (W4), and 2022 to 2023 (W7) (Table 2). Differences in prevalence of any tobacco, cigarette, e-product, cigarillo, filtered cigar, and pipe use were significantly higher among those with 1 or multiple disabilities compared with those with no disability in 2016 to 2018 and 2022 to 2023; while differences in cigar, hookah, and smokeless tobacco were minimal or nonsignificant. Prevalence in nearly all products similarly increased with number of disabilities in 2016 to 2018 and 2022 to 2023, and prevalence was consistently highest among those with 3 or more disabilities for all products. Magnitudes of differences remained stable or increased over time; for instance, cigarette use prevalence remained over 35% for those who experienced 3 or more disabilities across waves, while cigarette use among those with no disabilities ranged from 12.9% (95% CI, 12.3% to 13.5%) in 2022 to 2023 to 18.7% (95% CI, 18.2% to 19.3%) in 2013 to 2014, indicating that those who experienced 3 or more disabilities had a prevalence of cigarette use at least 1.9 times higher than those without a disability at each wave. Results for individual disabilities are presented in eTable 2 in Supplement 1.

**Table 2.** Weighted Age-Adjusted Prevalence of Tobacco Product Use Across Number of Disabilities and 3 Waves of Population Assessment of Tobacco and Health Study

Tobacco product	Wave 1 (September 2013 to December 2014)		Wave 4 (December 2016 to January 2018)		Wave 7 (January 2022 to April 2023)		Between-wave change in prevalence			
							Wave 4 to Wave 7		Wave 1 to Wave 7	
	No./Total No.^a^	Weighted % (95% CI)	No./Total No.^a^	Weighted % (95% CI)	No./Total No.^a^	Weighted % (95% CI)	Change (95% CI)	*P *value^b^	Change (95% CI)	*P *value^b^
Any tobacco product										
No. of disabilities										
0	11 534/23 115	25.4 (24.7 to 26.1)	10 528/24 914	23.9 (23.3 to 24.6)	7177/22 302	21.3 (20.5 to 22.1)	−2.6 (−3.2 to 2.0)	<.001	−4.0 (−4.7 to 3.4)	<.001
1	3166/4976	40.2 (38.6 to 41.8)	2957/5279	38.4 (36.7 to 40.2)	1959/4649	32.2 (30.3 to 34.2)	−6.2 (−7.9 to 4.6)	<.001	−8.0 (−10.1 to 5.8)	<.001
2	1336/1906	48.9 (45.4 to 52.5)	1241/1976	45.4 (42.2 to 48.5)	835/1737	40.4 (36.9 to 43.9)	−5.0 (−8.0 to 2.0)	<.001	−8.5 (−12.1 to 5.0)	<.001
≥3	1135/1605	51.8 (48.6 to 55.0)	946/1448	47.8 (44.2 to 51.5)	609/1073	47.2 (43.6 to 50.7)	−0.7 (−4.4 to 3.1)	.02	−4.6 (−8.7 to 0.6)	.02
Total No. of respondents	31 602	NA	33 617	NA	29 761	NA	NA	NA	NA	NA
Cigarettes										
No. of disabilities										
0	9210/23 588	18.7 (18.2 to 19.3)	7684/24 910	17.6 (17.1 to 18.2)	4014/22 295	12.9 (12.3 to 13.5)	−4.8 (−5.3 to 4.3)	<.001	−5.9 (−6.4 to 5.3)	<.001
1	2750/5072	32.9 (31.5 to 34.3)	2413/5279	31.4 (30.0 to 32.9)	1251/4648	22.2 (20.6 to 23.9)	−9.2 (−10.7 to 7.7)	<.001	−10.6 (−12.5 to 8.8)	<.001
2	1205/1947	42.3 (39.1 to 45.5)	1057/1976	39.2 (36.3 to 42.2)	584/1737	30.1 (26.8 to 33.7)	−9.1 (−12.4 to 5.7)	<.001	−12.2 (−16.0 to 8.3)	<.001
≥3	1041/1639	44.8 (41.5 to 48.2)	826/1448	41.1 (37.5 to 44.7)	483/1073	35.9 (32.4 to 39.6)	−5.1 (−8.8 to 1.5)	.006	−8.9 (−12.7 to 5.1)	<.001
Total No. of respondents	32 246	NA	33 613	NA	29 753	NA	NA	NA	NA	NA
E-products										
No. of disabilities										
0	2885/23 568	5.5 (5,2 to 5.8)	2731/24 895	5.2 (4.9 to 5.4)	3294/22 293	7.8 (7.4 to 8.2)	2.7 (2.2 to 3.1)	<.001	2.4 (1.9 to 2.8)	<.001
1	881/5063	10.3 (9.5 to 11.1)	776/5276	9.5 (8.7 to 10.4)	874/4647	12.0 (1.4 to 3.7)	2.5 (1.4 to 3.7)	<.001	1.7 (0.3 to 3.1)	.02
2	345/1942	12.3 (10.7 to 14.0)	331/1969	11.8 (10.4 to 13.3)	360/1734	14.5 (12.7 to 16.5)	2.8 (0.9 to 4.6)	.003	2.3 (0.3 to 4.3)	.02
≥3	316/1635	14.6 (12.6 to 17.0)	211/1446	12.4 (10.3 to 14.7)	210/1072	19.3 (16.4 to 22.6)	6.9 (3.6 to 10.3)	<.001	4.6 (1.0 to 8.3)	.01
Total No. of respondents	32 208	NA	33 586	NA	29 746	NA	NA	NA	NA	NA
Cigar										
No. of disabilities										
0	1532/23 388	3.3 (3.1 to 3.5)	1346/24 905	3.1 (2.9 to 3.4)	765/22 294	2.5 (2.2 to 2.8)	−0.6 (−0.9 to 0.3)	<.001	−0.8 (−1.1 to 0.5)	<.001
1	334/5015	4.2 (3.7 to 4.8)	312/5273	4.2 (3.6 to 4.8)	165/4647	2.9 (2.4 to 3.5)	−1.3 (−2.0 to 0.6)	<.001	−1.3 (−2.1 to 0.6)	<.001
2	135/1911	5.4 (4.4 to 6.7)	118/1973	4.3 (3.5 to 5.4)	68/1736	3.7 (2.7 to 5.0)	−0.7 (−1.9 to 0.6)	.29	−1.7 (−3.1 to 0.4)	.01
≥3	135/1607	6.1 (5.1 to 7.3)	88/1447	5.4 (4.2 to 6.9)	60/1073	6.1 (4.4 to 8.3)	0.7 (−1.5 to 2.9)	.54	0.0 (−2.2 to 2.1)	.97
Total No. of respondents	31 921	NA	33 598	NA	29 750	NA	NA	NA	NA	NA
Cigarillo										
No. of disabilities										
0	2100/22 951	3.7 (3.6 to 3.9)	1986/24 900	3.6 (3.4 to 3.8)	911/22 294	2.4 (2.1 to 2.6)	−1.2 (−1.5 to 0.9)	<.001	−1.4 (−1.7 to 1.1)	<.001
1	548/4885	6.2 (5.5 to 7.0)	554/5272	6.5 (5.8 to 7.3)	250/4645	3.6 (3.1 to 4.3)	−2.8 (−3.6 to 2.1)	<.001	−2.6 (−3.5 to 1.6)	<.001
2	198/1869	7.2 (6.2 to 8.3)	228/1973	7.7 (6.5 to 9.2)	118/1737	4.8 (3.8 to 6.1)	−2.9 (−4.4 to 1.3)	<.001	−2.4 (−3.8 to 0.9)	.002
≥3	210/1559	11.4 (9.7 to 13.4)	168/1443	9.9 (8.2 to 11.9)	90/1071	7.7 (5.9 to 9.8)	−2.2 (−4.8 to 0.4)	.09	−3.8 (−6.4 to 1.1)	.007
Total No. of respondents	31 264	NA	33 588	NA	29 747	NA	NA	NA	NA	NA
Filtered cigar										
No. of disabilities										
0	706/22 956	1.4 (1.2 to 1.5)	692/24 898	1.4 (1.2 to 1.5)	280/22 295	0.8 (0.7 to 0.9)	−0.6 (−0.7 to 0.4)	<.001	−0.5 (−0.7 to 0.4)	<.001
1	247/4887	2.9 (2.5 to 3.3)	252/5275	3.0 (2.5 to 3.5)	103/4645	1.6 (1.2 to 2.1)	−1.4 (−1.9 to 0.8)	<.001	−1.3 (−1.8 to 0.7)	<.001
2	106/1868	3.9 (3.2 to 4.7)	105/1973	3.7 (2.8 to 4.8)	48/1737	1.9 (1.3 to 2.9)^c^	−1.7 (−2.9 to 0.6)	.003	−1.9 (−3.0 to 0.9)	<.001
≥3	126/1560	5.9 (4.6 to 7.6)	115/1445	6.0 (4.8 to 7.6)	53/1069	4.3 (3.0 to 6.2)	−1.8 (−3.7 to 0.1)	.07	−1.6 (−3.4 to 0.3)	.09
Total No. of respondents	31 271	NA	33 591	NA	29 746	NA	NA	NA	NA	NA
Pipe										
No. of disabilities										
0	347/23 570	0.7 (0.6 to 0.8)	287/24 905	0.6 (0.5 to 0.7)	137/22 296	0.4 (0.4 to 0.5)	−0.2 (−0.3 to 0.0)	.02	−0.3 (−0.4 to 0.1)	<.001
1	117/5064	1.2 (0.9 to 1.4)	107/5278	1.3 (1.0 to 1.6)	41/4649	0.7 (0.4 to 1.0)^c^	−0.6 (−1.0 to 0.3)	<.001	−0.5 (−0.8 to 0.2)	.004
2	53/1942	1.8 (1.3 to 2.4)	43/1976	1.5 (1.0 to 2.2)^c^	18/1737	0.7 (0.4 to 1.2)^c^	−0.8 (−1.5 to 0.2)	.02	−1.1 (−1.7 to 0.4)	<.001
≥3	66/1633	3.2 (2.4 to 4.4)	32/1446	2.4 (1.4 to 4.0)^c^	25/1072	2.9 (1.6 to 5.3)^c^	0.5 (−1.7 to 2.6)	.67	−0.4 (−2.3 to 1.6)	.71
Total No. of respondents	32 209	NA	33 605	NA	29 754	NA	NA	NA	NA	NA
Hookah										
No. of disabilities										
0	1276/23 581	2.0 (1.9 to 2.2)	883/24 907	1.6 (1.5 to 1.8)	500/22 295	1.2 (1.1 to 1.4)	−0.4 (−0.6 to 0.2)	<.001	−0.8 (−1.0 to 0.6)	<.001
1	283/5065	2.6 (2.3 to 3.0)	233/5277	2.5 (2.1 to 2.9)	97/4646	1.3 (1.0 to 1.7)	−1.1 (−1.6 to 0.6)	<.001	−1.3 (−1.8 to 0.8)	<.001
2	101/1943	3.3 (2.7 to 4.0)	76/1973	2.3 (1.8 to 2.8)	38/1737	1.8 (1.1 to 2.9)^c^	−0.5 (−1.4 to 0.4)	.25	−1.5 (−2.7 to 0.3)	.01
≥3	59/1633	3.7 (2.7 to 4.9)	49/1447	3.1 (2.2 to 4.3)^c^	25/1072	2.5 (1.4 to 4.4)^c^	−0.5 (−2.2 to 1.1)	.52	−1.1 (−2.9 to 0.6)	.19
Total No. of respondents	32 222	NA	33 604	NA	29 750	NA	NA	NA	NA	NA
Smokeless										
No. of disabilities										
0	1360/23 398	2.8 (2.6 to 3.0)	1171/24 906	2.8 (2.6 to 3.0)	489/22 293	2.0 (1.7 to 2.2)	−0.8 (−1.0 to 0.6)	<.001	−0.9 (−1.1 to 0.7)	<.001
1	278/5026	3.6 (3.1 to 4.1)	237/5278	3.6 (2.9 to 4.3)	107/4648	2.4 (1.9 to 3.0)	−1.2 (−1.7 to 0.6)	<.001	−1.1 (−1.7 to 0.6)	<.001
2	94/1925	3.5 (2.8 to 4.3)	102/1975	4.2 (3.3 to 5.4)	41/1736	2.5 (1.8 to 3.5)^c^	−1.7 (−2.8 to 0.6)	.002	−0.9 (−1.9 to 0.1)	.07
≥3	89/1615	3.9 (3.0 to 5.0)	74/1446	4.7 (3.4 to 6.5)	38/1071	3.8 (2.8 to 5.2)^c^	−0.9 (−2.8 to 1.0)	.34	−0.1 (−1.7 to 1.5)	.91
Total No. of respondents	31 964	NA	33 605	NA	29 748	NA	NA	NA	NA	NA

Abbreviation: NA, not applicable.

^a^ Represents unweighted sample size for the numerator for each corresponding estimate with total unweighted number as the denominator.

^b^ Significance tests are based on design-adjusted statistical tests for change across waves within number of disability categories.

^c^ This is flagged because the estimate has low precision due to sample size.

### Differences in Tobacco Use Prevalence Across Waves

Even though differences in tobacco use associated with cumulative disabilities remained stable over time, tobacco use prevalence, aside from e-product use, declined from 2013 to 2014 (W1) to 2022 to 2023 (W7) and from 2016 to 2018 (W4) to 2022 to 2023 (W7) among all individuals (Table 2). However, changes in tobacco use prevalence over time were not statistically significant for all products for the group with 3 or more disabilities. For the group with 3 or more disabilities, changes in prevalence from 2013 to 2014 to 2022 to 2023 for cigar (0.0%; 95% CI, −2.2% to 2.1%), filtered cigar (−1.6%; 95% CI, −3.4% to 0.3%), pipe (−0.4%; 95% CI, −2.3% to 1.6%), hookah (−1.1%; 95% CI, −2.9% to 0.6%), and smokeless tobacco (−0.1%; 95% CI, −1.7% to 1.5%) were not statistically significant. The declines in any tobacco (−4.6%; 95% CI, −8.7% to −0.6%) and cigarette (−8.9%; 95% CI, −12.7% to −5.1%) use were small, and increases in e-product use were large (4.6%; 95% CI, 1.0% to 8.3%) for those with 3 or more disabilities compared with other groups. Additionally, changes in prevalence from 2016 to 2018 to 2022 to 2023 for any tobacco (−0.7%; 95% CI, −4.4% to 3.1%), cigar (0.7%; 95% CI, −1.5% to 2.9%), cigarillo (−2.2%; 95% CI, −4.8% to 0.4%), filtered cigar (−1.8%; 95% CI, −3.7% to 0.1%), pipe (0.5%; 95% CI, −1.7% to 2.6%), hookah (−0.5%; 95% CI, −2.2% to 1.1%), and smokeless tobacco (−0.9%; 95% CI, −2.8% to 1.0%) were not statistically significant. The lack of change in any tobacco use from 2016 to 2018 to 2022 to 2023 was potentially due to e-product use. In supplemental analyses that excluded e-product use, changes in any tobacco use from 2013 to 2014 (50.5%; 95% CI, 47.3% to 53.7%) to 2022 to 2023 (39.9%; 95% CI, 36.4% to 43.4%) and from 2016 to 2018 (46.4%; 95% CI, 42.9% to 50.0%) to 2022 to 2023 were statistically significant (respectively, −10.6%; 95% CI, −14.5% to −6.7% and −6.5%; 95% CI, −10.1% to −2.9%) for those with 3 or more disabilities. Those with 2 disabilities had the largest absolute decline in use for any tobacco, cigarettes, cigars, filtered cigar, pipe, or hookah, followed by those with only 1 disability (Table 2). Those with 3 or more disabilities had the largest absolute decline in cigarillos, and the largest absolute increase in e-product use.

As shown in eTable 3 in Supplement 1, supplemental analyses showed that the interactions between time and number of disabilities were statistically significant for any tobacco use (*F* score = 5.64; *P* = <.001), cigarette use (*F* score = 5.05; *P* = <.001), e-product use (*F* score = 2.94; *P* = .01), and smokeless tobacco use (*F* score = 5.07; *P* = <.001). These findings indicate that changes in use prevalence over time for these products significantly varied according to cumulative disabilities.

## Discussion

Using data from the PATH Study, we found marked differences in tobacco use across number of disabilities. Prevalence of use for nearly every tobacco product, except for cigars, hookah, and smokeless tobacco, was positively associated with cumulative disabilities—prevalence increased with number of disabilities. This finding corresponds with previous research showing tobacco use is notably higher among individuals who experience comorbidity.^6,7,18,25,35^ This study extends this literature by showing how comorbidity defined as cumulative disabilities is a critical dimension of current tobacco-related disparities in the US.

A second key finding is that prevalence was consistently most dramatic for those who experienced the highest number of disabilities (≥3) compared with those with no disabilities (and generally those with 1 or 2 disabilities). Individuals with multiple disabilities were most at risk for tobacco use—the leading preventable cause of mortality and morbidity in the US.^1,2,3,4^ IWD who use tobacco are more vulnerable to tobacco-related health risks relative to those without disabilities, and they are more likely to experience exacerbated health conditions associated with their disabilities due to tobacco use.^36^ IWD are at risk for secondary conditions linked to their primary disabilities,^37,38^ and individuals with more disabilities are more at risk for secondary conditions. Future research should consider how tobacco use among those with cumulative disabilities influences future morbidity or mortality, and how tobacco use is linked to secondary conditions. Finally, we found differences in tobacco use associated with cumulative disabilities were stable over time (and even got worse for some tobacco products), despite moderate declines in tobacco use among IWD. Moreover, recent increases in e-product use were most dramatic among IWD, and especially those with 3 or more disabilities.

IWD who use tobacco are more vulnerable to health risks relative to those without disabilities, in part due to limited access to health resources.^36,39,40^ Additionally, tobacco use may contribute to the development of disabilities and therefore generate additional health problems,^36^ making prevention and cessation efforts among IWD a priority. Despite higher prevalence of tobacco use and greater health risks, IWD are less likely to attempt, and succeed at, quitting tobacco compared with individuals without a disability^39,41^ Moreover, IWD are more likely to experience other issues that could exacerbate tobacco-related health disparities (eg, lower socioeconomic status), and these intersectional characteristics can potentially influence how they receive and use cessation materials.^39^

Research on cessation assistance received by IWD has shown mixed results to date: some studies have shown that IWD who smoke have received higher rates of referrals to cessation services from health care clinicians compared with those without a disability who smoke cigarettes,^36,42,43^ while other studies have shown lower rates of cessation assistance or referrals.^36^ Additionally, individuals with physical and mental health comorbidities have the highest rates of tobacco use, but they do not receive disproportionately higher rates of screening or cessation advice needed to reduce disparities.^44^ Previous research has not assessed cumulative disabilities and tobacco use screening or cessation advice receipt from clinicians, or how cumulative disabilities are associated with barriers to tobacco cessation. Previous research has primarily addressed how health care clinicians target cigarette smoking, but less is known about how clinicians address noncigarette tobacco use.^44^ Future research should assess how screening and cessation (for all products) are associated with cumulative disabilities. Future studies could consider clinician-level interventions geared toward training health care professionals on how best to provide cessation services to those with cumulative disabilities.^36^ Barriers to treatment may contribute to stable tobacco-related disparities over time, and future work is needed to determine how to provide the tools needed to address these barriers.

### Limitations

This study had limitations. The PATH Study was designed to provide valid estimates of tobacco use over time. However, individuals can develop disabilities over time, and prevalence of disabilities at future waves could be an underestimate of the actual population prevalence. Additionally, there were higher rates of study attrition among those with a disability. Thus, based on these limitations, conclusions should not be drawn about change in disability prevalence over time. This study focused on cross-sectional associations of individual products, and future research should assess the temporal ordering of the disability-tobacco use relationship, and how severity of use varies across disabilities.

## Conclusions

In this study, individuals with multiple disabilities were more likely to use tobacco products compared with those with no or fewer disabilities. These disparities in tobacco use did not change over time, despite marked declines in tobacco use over time. IWD are more likely to experience health risks associated with tobacco use, and research should assess how tobacco use among those with multiple disabilities affects future health status. More research is needed on how best to provide cessation services, and especially services that go beyond cessation of cigarettes, to those who experience cumulative disabilities.

## Notes

Supplement 1. **eTable 1.** Significance Tests for Within-wave Differences Across Number of Disability Categories for Each Tobacco Use Measure: Waves 1, 4, and 7 **eTable 2.** Weighted and Age-Adjusted Prevalence of Tobacco Produce Use Across Any Disability and Separate Disabilities Within Waves **eTable 3.** Weighted Logistic Regression Results for Tobacco and E-product Use and Associations With Disabilities from 2013−2014 (Wave 1) to 2022-2023 (Wave 7) of the Population Assessment of Tobacco and Health Study **eMethods.** Methods for Supplemental Analyses in eTable

Supplement 2.
Data Sharing Statement
