# Supplementary material for: Tobacco and E-Product Use by US Adults With Disabilities
Source: JAMA Netw Open. 2025 Feb 18;8(2):e2460471. doi: 10.1001/jamanetworkopen.2024.60471 (PMC11836758; doi:10.1001/jamanetworkopen.2024.60471)
Supplement: Supplement 1. — eTable 1. Significance Tests for Within-wave Differences Across Number of Disability Categories for Each Tobacco Use Measure: Waves 1, 4, and 7 eTable 2. Weighted and Age-Adjusted Prevalence of Tobacco Produce Use Across Any Disability and Separate Disabilities Within Waves eTable 3. Weighted Logistic Regression Results for Tobacco and E-product Use and Associations With Disabilities from 2013−2014 (Wave 1) to 2022-2023 (Wave 7) of the Population Assessment of Tobacco and Health Study eMethods. Methods for Supplemental Analyses in eTable [file jamanetwopen-e2460471-s001.pdf]

Supplemental Online Content

Parks MJ, Marshall D, Kimmel HL, et al. Tobacco and e-product use by US adults with disabilities. *JAMA Netw Open*. 2025;8(2):e2460471. doi:10.1001/jamanetworkopen.2024.60471

eTable1. Significance Tests for Within-wave Differences Across Number of Disability Categories for Each Tobacco Use Measure: Waves 1, 4, and 7

eTable2. Weighted and Age-adjusted Prevalence of Tobacco Product Use Across Any Disability and Separate Disabilities Within Waves

eTable3. Weighted Logistic Regression Results for Tobacco and E-product Use and Associations with Disabilities from 2013/2014 (Wave 1) to 2022/2023 (Wave 7) of the Population Assessment of Tobacco and Health Study

eMethods. Methods for supplemental analyses in eTable3

This supplemental material has been provided by the authors to give readers additional information about their work.

© 2025 Parks MJ et al. *JAMA Network Open*.

eTable 1. Significance Tests for Within-wave Differences Across Number of Disability Categories for Each Tobacco Use Measure, Waves 1, 4, and 7

Tobacco product	Wave 1 (September 2013 to December 2014)		Wave 4 (December 2016 to January 2018)		Wave 7 (January 2022 to April 2023)	
	Design-adjusted statistical test	*P* value	Design-adjusted statistical test	*P* value	Design-adjusted statistical test	*P* value
Any tobacco product	95.5	.001	107.9	.001	96.8	.001
Cigarettes	149.6	.001	152.2	.001	118.0	.001
E-products	49.4	.001	44.4	.001	31.8	.001
Cigar	3.3	.02	1.3	.28	6.9	.001
Cigarillo	14.7	.001	21.5	.001	21.2	.001
Filtered cigar	49.4	.001	53.9	.001	31.0	.001
Pipe	22.4	.001	13.0	.001	12.6	.001
Hookah	2.9	.04	1.9	.14	0.8	.49
Smokeless	0.5	.65	2.5	.06	3.1	.03

© 2025 Parks MJ et al. *JAMA Network Open*.

eTable 2: Weighted and Age-adjusted Prevalence of Tobacco Product Use Across Any Disability and Separate Disabilities Within Waves

	Wave 1 (9/2013 - 12/2014)					Wave 4 (12/2016 - 1/2018)					Wave 7 (1/2022 - 4/2023)				
	%	95% CI	%	95% CI	Sig. test	%	95% CI	%	95% CI	Sig. test	%	95% CI	%	95% CI	Sig. test
Any Disability		Yes		No			Yes		No			Yes		No	
Any tobacco product	44.0	(42.5, 45.4)	25.4	(24.7, 26.1)	315.6 ***	36.5	(35.3, 37.8)	24.9	(24.2, 25.6)	267.8 ***	36.0	(34.6, 37.5)	21.3	(20.5, 22.1)	226.6 ***
Cigarettes	36.8	(35.4, 38.2)	18.7	(18.2, 19.3)	434.0 ***	34.4	(33.2, 35.7)	17.6	(17.1, 18.2)	419.9 ***	25.7	(24.4, 27.1)	12.9	(12.3, 13.5)	236.6 ***
E-products	11.4	(10.6,.12.1)	5.5	(5.2, 5.8)	132.7 ***	10.3	(9.6, 11.1)	5.2	(4.9, 5.4)	120.0 ***	13.4	(12.5, 14.3)	7.8	(7.4, 8.2)	70.2 ***
Cigar	4.7	(4.3, 5.2)	3.3	(3.1, 3.5)	9.1 **	4.4	(3.9, 4.9)	3.1	(2.9, 3.4)	3.2	3.5	(3.0, 4.0)	2.5	(2.2, 2.8)	4.3 *
Cigarillo	7.1	(6.5, 7.8)	3.7	(3.6, 3.9)	37.0 ***	7.2	(6.6, 7.9)	3.6	(3.4, 3.8)	59.2 ***	4.4	(3.9, 5.0)	2.4	(2.1, 2.6)	30.9 ***
Filtered cigar	3.6	(3.2, 4.0)	1.4	(1.2,.1.5)	146.2 ***	3.6	(3.2, 4.1)	1.4	(1.2, 1.5)	133.1 ***	2.0	(1.7, 2.5)	0.8	(0.7, 0.9)	53.3 ***
Pipe	1.6	(1.4, 1.8)	0.7	(0.6, 0.8)	47.4 ***	1.5	(1.2, 1.8)	0.6	(0.5, 0.7)	36.5 ***	0.9	(0.7, 1.2)	0.4	(0.4, 0.5)	10.5 **
Hookah	2.9	(2.6, 3.2)	2.0	(1.9, 2.2)	0.6	2.5	(2.2, 2.9)	1.6	(1.5, 1.8)	1.5	1.6	(1.3, 2.0)	1.2	(1.2, 1.5)	0.1
Smokeless	3.6	(3.2, 3.9)	2.8	(2.6, 3.0)	0.8	3.9	(3.4, 4.4)	2.8	(2.6, 3.0)	6.5 *	2.6	(2.2, 3.0)	2.0	(1.7, 2.2)	3.6
Mobility		Yes		No			Yes		No			Yes		No	
Any tobacco product	49.2	(47.1, 51.4)	27.1	(26.4, 27.8)	114.2 ***	45.9	(43.6, 48.2)	25.7	(25.0, 26.3)	74.8 ***	42.9	(40.4, 45.4)	22.7	(22.0, 23.4)	118.6 ***
Cigarettes	42.0	(40.0, 44.1)	20.3	(19.8, 21.0)	169.2 ***	39.6	(37.6, 41.7)	19.2	(18.7, 19.8)	136.9 ***	33.0	(30.7, 35.4)	14.0	(13.4, 14.5)	254.0 ***
E-products	12.6	(11.4, 14.0)	6.0	(5.8, 6.3)	38.7 ***	10.9	(9.8, 12.1)	5.8	(5.5, 6.0)	4.2 *	14.9	(13.2, 16.8)	8.5	(8.1, 8.9)	0.7
Cigar	5.5	(4.8, 6.3)	3.4	(3.2, 3.6)	0.9	4.8	(4.0, 5.7)	3.3	(3.1, 3.5)	0.3	3.7	(2.9, 4.7)	2.6	(2.4, 2.8)	0.6
Cigarillo	8.9	(7.8, 10.1)	4.0	(3.9, 4.2)	1.2	9.1	(8.0, 10.3)	3.9	(3.7, 4.1)	6.5 *	5.5	(4.5, 6.6)	2.6	(2.5, 2.9)	9.6 **
Filtered cigar	4.3	(3.5, 5.2)	1.5	(1.4, 1.7)	65.7 ***	4.3	(3.6, 5.1)	1.6	(1.5, 1.7)	61.8 ***	2.9	(2.2, 3.9)	0.9	(0.8, 1.0)	45.0 ***
Pipe	2.0	(1.7, 2.5)	0.8	(0.7, 0.9)	21.0 ***	1.7	(1.2, 2.3)	0.7	(0.6, 0.8)	5.2 *	1.3	(0.8, 2.3)	0.5	(0.4, 0.6)	4.4 *
Hookah	3.5	(2.9, 4.2)	2.1	(2.0, 2.3)	35.1 ***	3.0	(2.4, 3.6)	1.7	(1.6, 1.9)	11.1 **	2.5	(1.8, 3.6)	1.2	(1.1, 1.4)	0.1
Smokeless	3.7	(3.2, 4.4)	2.9	(2.7, 3.1)	0.5	3.7	(2.9, 4.7)	2.9	(2.7, 3.1)	0.0	2.7	(2.1, 3.5)	2.0	(1.8, 2.2)	2.0
Independent Living		Yes		No			Yes		No			Yes		No	
Any tobacco product	47.0	(44.1, 50.0)	28.4	(28.0, 29.1)	66.9 ***	43.7	(40.8, 46.7)	26.8	(26.2, 27.4)	85.6 ***	40.9	(38.1, 43.9)	23.6	(22.9, 24.3)	158.2 ***
Cigarettes	41.2	(38.4, 44.1)	21.5	(21.0, 22.1)	137.5 ***	37.5	(34.7, 40.4)	20.3	(19.8, 20.9)	130.6 ***	31.2	(28.2, 34.3)	14.8	(14.3, 15.4)	136.1 ***
E-products	12.7	(11.1, 14.4)	6.4	(6.1, 6.7)	34.2 ***	11.9	(10.3, 13.7)	5.9	(5.7, 6.2)	43.5 ***	16.4	(14.8, 18.2)	8.6	(8.2, 8.9)	126.7 ***
Cigar	5.4	(4.4, 6.5)	3.5	(3.3, 3.7)	4.2 *	4.4	(3.5, 5.5)	3.3	(3.1, 3.5)	1.0	4.1	(2.9, 5.6)	2.6	(2.4, 2.9)	5.2 *
Cigarillo	8.7	(7.6, 9.9)	4.2	(4.0, 4.4)	30.3 ***	7.9	(6.7, 9.3)	4.1	(3.9, 4.3)	28.6 ***	5.6	(4.5, 7.1)	2.6	(2.4, 2.9)	30.5 ***
Filtered cigar	4.9	(4.1, 5.9)	1.6	(1.5, 1.8)	75.2 ***	4.6	(3.8, 5.6)	1.7	(1.6, 1.8)	85.2 ***	2.8	(2.0, 3.8)	1.0	(0.9, 1.1)	40.8 ***
Pipe	2.0	(1.6, 2.6)	0.8	(0.7, 0.9)	21.2 ***	2.0	(1.3, 3.1)	0.7	(0.7, 0.8)	17.5 ***	1.6	(0.9, 2.8)	0.5	(0.4, 0.6)	16.5 ***
Hookah	3.2	(2.5, 3.9)	2.2	(2.0, 2.3)	0.4	2.3	(1.8, 2.9)	1.8	(1.6, 1.9)	0.2	1.3	(0.7, 2.2)	1.3	(1.2, 1.4)	0.0
Smokeless	3.3	(2.8, 4.0)	3.0	(2.8, 3.2)	0.2	4.5	(3.5, 5.7)	2.9	(2.7, 3.1)	4.5 *	2.6	(1.8, 3.5)	2.1	(1.8, 2.3)	0.6
Self-care		Yes		No			Yes		No			Yes		No	
Any tobacco product	45.4	(41.4, 49.5)	28.9	(28.2, 29.6)	28.2 ***	41.7	(37.7, 45.9)	27.3	(26.7, 27.9)	15.8 ***	41.5	(36.4, 46.7)	24.0	(23.3, 24.8)	39.2 ***
Cigarettes	37.2	(33.6, 40.9)	22.1	(21.5, 22.7)	51.4 ***	36.0	(32.1, 40.1)	20.8	(20.3, 21.4)	29.8 ***	31.5	(26.8, 36.5)	15.3	(14.7, 15.8)	58.4 ***
E-products	11.4	(9.3, 13.9)	6.5	(6.2, 6.8)	8.7 **	10.1	(8.3, 12.3)	6.1	(5.9, 6.4)	1.1	15.9	(12.0, 20.7)	8.8	(8.4, 9.2)	4.3 *
Cigar	5.0	(4.1, 6.2)	3.5	(3.3, 3.7)	1.6	4.4	(3.2, 6.0)	3.3	(3.1, 3.5)	0.0	5.6	(3.5, 9.0)	2.6	(2.4, 2.9)	5.6 *
Cigarillo	8.8	(7.2, 10.7)	4.3	(4.1, 4.5)	3.4	8.5	(6.4, 11.3)	4.2	(4.0, 4.4)	2.5	6.7	(4.4, 10.1)	2.7	(2.5, 2.9)	10.6 **
Filtered cigar	3.8	(2.7, 5.2)	1.8	(1.6, 1.9)	12.3 ***	5.3	(3.8, 7.3)	1.8	(1.6, 1.9)	20.5 ***	2.8	(1.7, 4.5)	1.0	(0.9, 1.2)	21.7 ***
Pipe	2.7	(1.8, 4.1)	0.8	(0.8, 0.9)	15.0 ***	2.6	(1.5, 4.5)	0.7	(0.7, 0.8)	8.1 **	2.9	(1.2, 6.8)	0.5	(0.4, 0.6)	8.5 **
Hookah	3.9	(2.8, 5.3)	2.2	(2.0, 2.4)	2.6	3.3	(2.4, 4.7)	1.8	(1.6, 1.9)	0.1	3.1	(1.6, 6.0)	1.3	(1.2, 1.4)	0.9
Smokeless	3.7	(2.7, 5.0)	3.0	(2.8, 3.2)	0.1	4.6	(2.9, 7.1)	3.0	(2.8, 3.2)	2.0	4.7	(3.1, 6.9)	2.0	(1.8, 2.3)	17.2 ***
Hearing		Yes		No			Yes		No			Yes		No	
Any tobacco product	44.1	(40.3, 48.0)	28.9	(28.2, 29.7)	0.1	38.8	(35.3, 42.4)	27.3	(26.7, 27.9)	0.1	38.1	(34.0, 42.4)	24.1	(23.4, 24.8)	3.7
Cigarettes	34.6	(31.4, 40.0)	22.2	(21.6, 22.9)	0.2	30.3	(26.9, 33.9)	20.9	(20.4, 21.5)	0.3	26.0	(22.5, 29.7)	15.3	(14.8, 15.9)	7.8 **
E-products	11.6	(9.9, 13.6)	6.5	(6.2, 6.8)	0.5	9.8	(8.0, 11.9)	6.1	(5.9, 6.4)	2.5	13.9	(11.4, 16.8)	8.8	(8.5, 9.2)	2.0
Cigar	6.0	(4.9, 7.3)	3.4	(3.3, 3.6)	3.4	5.5	(4.2, 7.3)	3.3	(3.1, 3.5)	0.5	5.3	(3.8, 7.4)	2.6	(2.4, 2.8)	3.8
Cigarillo	7.2	(6.0, 8.7)	4.3	(4.1, 4.5)	5.4 *	7.6	(5.9, 9.6)	4.2	(4.0, 4.4)	0.2	5.2	(3.8, 7.2)	2.7	(2.5, 2.9)	0.9
Filtered cigar	2.9	(2.2, 3.9)	1.8	(1.6, 1.9)	1.2	4.0	(2.9, 5.4)	1.8	(1.6, 1.9)	9.4 **	2.0	(1.2, 3.3)	1.1	(0.9, 1.2)	0.9
Pipe	2.8	(2.0, 3.9)	0.8	(0.7, 0.9)	15.5 ***	2.6	(1.4, 4.7)	0.7	(0.7, 0.8)	9.5 **	2.4	(1.3, 4.3)	0.5	(0.4, 0.6)	16.5 ***
Hookah	3.5	(2.6, 4.8)	2.2	(2.0, 2.4)	15.6 ***	2.2	(1.5, 3.3)	1.8	(1.7, 1.9)	12.0 ***	2.6	(1.3, 5.1)	1.3	(1.2, 1.4)	0.0
Smokeless	7.0	(5.3, 9.1)	2.9	(2.7, 3.1)	9.3 **	6.2	(4.6, 8.5)	2.9	(2.7, 3.1)	10.2 **	4.5	(3.2, 6.4)	2.0	(1.8, 2.2)	8.8 **
Vision		Yes		No			Yes		No			Yes		No	
Any tobacco product	46.0	(43.1, 48.9)	28.4	(27.7, 29.1)	128.8 ***	44.9	(42.2, 47.6)	26.7	(26.1, 27.3)	143.1 ***	38.3	(35.0, 41.7)	23.8	(23.1, 24.4)	84.1 ***
Cigarettes	40.0	(36.2, 41.8)	21.6	(21.0, 22.2)	181.5 ***	37.9	(35.4, 40.5)	20.3	(19.8, 20.8)	206.1 ***	27.1	(24.4, 29.8)	15.0	(14.5, 15.5)	120.5 ***
E-products	11.3	(10.0, 12.7)	6.4	(6.1, 6.7)	41.8 ***	10.3	(9.0, 11.9)	6.0	(5.8, 6.3)	33.9 ***	13.6	(11.8, 15.5)	8.7	(8.4, 9.1)	19.6 ***
Cigar	4.6	(3.8, 5.5)	3.5	(3.3, 3.7)	1.3	5.0	(3.9, 6.3)	3.3	(3.1, 3.5)	2.5	3.8	(2.8, 5.0)	2.6	(2.4, 2.9)	3.2
Cigarillo	8.0	(6.8, 9.4)	4.2	(4.0, 4.4)	34.4 ***	7.6	(6.5, 8.9)	4.1	(3.9, 4.3)	29.2 ***	4.9	(3.9, 6.3)	2.7	(2.5, 2.9)	14.8 ***
Filtered cigar	3.7	(2.9, 4.7)	1.7	(1.6, 1.9)	52.4 ***	4.9	(4.0, 6.0)	1.7	(1.6, 1.8)	92.1 ***	2.0	(1.4, 2.8)	1.0	(0.9, 1.2)	13.6 ***
Pipe	1.6	(1.2, 2.2)	0.8	(0.7, 0.9)	14.6 ***	1.8	(1.1, 2.8)	0.7	(0.7, 0.8)	8.0 **	1.2	(0.8, 1.9)	0.5	(0.4, 0.6)	7.7 **
Hookah	2.6	(2.1, 3.3)	2.2	(2.0, 2.4)	0.1	2.9	(2.2, 3.8)	1.8	(1.6, 1.9)	3.4	2.1	(1.2, 3.3)	1.3	(1.1, 1.4)	2.2
Smokeless	2.8	(2.1, 3.7)	3.0	(2.8, 3.2)	0.7	3.7	(2.8, 5.1)	3.0	(2.8, 3.2)	1.3	2.8	(1.9, 4.2)	2.1	(1.8, 2.3)	1.9
Cognitive		Yes		No			Yes		No			Yes		No	
Any tobacco product	47.1	(44.3, 50.0)	27.6	(27.0, 28.3)	180.8 ***	43.8	(41.5, 46.2)	26.0	(25.4, 26.6)	257.2 ***	38.9	(36.4, 41.4)	22.9	(22.2, 23.6)	215.9 ***
Cigarettes	40.7	(38.1, 43.4)	20.8	(20.2, 21.3)	259.0 ***	37.4	(35.3, 39.6)	19.5	(19.0, 20.0)	325.3 ***	28.5	(26.2, 31.0)	14.2	(13.7, 14.8)	194.0 ***
E-products	13.3	(12.1, 14.5)	6.0	(5.8, 6.3)	220.2 ***	11.6	(10.5, 12.8)	5.7	(5.4, 5.9)	228.2 ***	14.6	(13.5, 15.8)	8.2	(7.9, 8.6)	209.6 ***
Cigar	5.3	(4.5, 6.1)	3.4	(3.2, 3.6)	24.0 ***	4.6	(3.8, 5.5)	3.2	(3.0, 3.4)	10.9 **	4.0	(3.3, 4.9)	2.6	(2.3, 2.8)	13.7 ***
Cigarillo	7.9	(7.0, 8.8)	4.0	(3.9, 4.2)	118.2 ***	7.6	(6.7, 8.7)	3.9	(3.8, 4.1)	112.1 ***	5.1	(4.3, 6.0)	2.5	(2.3, 2.8)	60.9 ***
Filtered cigar	4.6	(4.0, 5.4)	1.5	(1.4, 1.7)	205.3 ***	4.3	(3.6, 5.1)	1.6	(1.5, 1.7)	138.4 ***	2.7	(2.0, 3.5)	0.9	(0.8, 1.0)	51.5 ***
Pipe	2.0	(1.6, 2.4)	0.8	(0.7, 0.9)	85.3 ***	1.5	(1.2, 2.1)	0.7	(0.6, 0.8)	24.4 ***	0.9	(0.6, 1.4)	0.5	(0.4, 0.6)	8.8 **
Hookah	3.1	(2.7, 3.6)	2.1	(1.9, 2.3)	24.9 ***	2.6	(2.1, 3.2)	1.7	(1.6, 1.8)	21.1 ***	1.6	(1.2, 2.1)	1.3	(1.1, 1.4)	5.2 *
Smokeless	3.4	(3.0, 3.8)	3.0	(2.8, 3.2)	1.2	3.9	(3.3, 4.5)	2.9	(2.7, 3.2)	9.7 **	2.4	(2.0, 3.0)	2.0	(1.8, 2.3)	1.1

Significance tests are based on design-adjusted F-tests across disability type categories (yes/no) within waves.
N for Wave 1 range: 31201 to 32246; N for Wave 4 range: 33506 to 33617; N for Wave 7 range: 29685 to 29761.
*=p<.05; **=p<.01; ***=p<.001; all differences with p<.05 were non-significant after adjusting for multiple

© 2025 Parks MJ et al. *JAMA Network Open*.

eTable 3. Weighted Logistic Regression Results for Tobacco and E-product Use and Associations with Disabilities from 2013/2014 (Wave 1) to 2022/2023 (Wave 7) of the Population Assessment of Tobacco and Health Study

	Any Tobacco Use			Cigarette Use			E-Product Use			Cigar Use			Cigarillo Use			Filtered Cigar Use			Pipe Use			Hookah Use			Smokeless Product Use		
Variables	Log-odds	S.E.	P-value	Log-odds	S.E.	P-value	Log-odds	S.E.	P-value	Log-odds	S.E.	P-value	Log-odds	S.E.	P-value	Log-odds	S.E.	P-value	Log-odds	S.E.	P-value	Log-odds	S.E.	P-value	Log-odds	S.E.	P-value
Wave (year)																											
Wave 1 (2013/2014; reference)	~	~	~	~	~	~	~	~	~	~	~	~	~	~	~	~	~	~	~	~	~	~	~	~	~	~	~
Wave 4 (2016/2018)	-0.06	(0.01)	***	-0.06	(0.01)	***	-0.03	(0.03)		-0.03	(0.04)		-0.03	(0.03)		0.03	(0.06)		-0.13	(0.09)		-0.21	(0.05)	***	-0.01	(0.03)	
Wave 7 (2022/2023)	-0.20	(0.02)	***	-0.43	(0.02)	***	0.46	(0.04)	***	-0.25	(0.06)	***	-0.43	(0.06)	***	-0.48	(0.08)	***	-0.40	(0.12)	**	-0.44	(0.07)	***	-0.36	(0.05)	***
Cumulative Disability																											
0 (reference)	~	~	~	~	~	~	~	~	~	~	~	~	~	~	~	~	~	~	~	~	~	~	~	~	~	~	~
1	0.70	(0.04)	***	0.74	(0.03)	***	0.74	(0.05)	***	0.25	(0.07)	***	0.54	(0.07)	***	0.76	(0.09)	***	0.55	(0.12)	***	0.32	(0.08)	***	1.70	(1.96)	**
2	1.02	(0.07)	***	1.08	(0.06)	***	1.00	(0.08)	***	0.47	(0.11)	***	0.67	(0.09)	***	1.07	(0.12)	***	0.90	(0.18)	***	0.48	(0.11)	***	1.70	(1.96)	
3+	1.10	(0.06)	***	1.15	(0.06)	***	1.25	(0.08)	***	0.58	(0.11)	***	1.18	(0.09)	***	1.51	(0.13)	***	1.39	(0.16)	***	0.53	(0.17)	**	1.70	(1.96)	**
Wave X cumulative disability interactio	~	~	~	~	~	~	~	~	~	~	~	~	~	~	~	~	~	~	~	~	~	~	~	~	~	~	~
Wave 4 X 1 disability	0.00	(0.04)		0.02	(0.04)		-0.06	(0.07)		0.04	(0.10)		0.08	(0.08)		0.03	(0.11)		0.26	(0.18)		1.70	(1.96)		1.70	(1.96)	
Wave 4 X 2 disabilities	-0.06	(0.05)		-0.01	(0.05)		-0.07	(0.10)		-0.22	(0.13)		0.13	(0.12)		-0.06	(0.16)		-0.10	(0.26)		1.70	(1.96)		1.70	(1.96)	*
Wave 4 X 3+ disabilities	-0.05	(0.06)		-0.02	(0.06)		-0.20	(0.12)		-0.12	(0.21)		-0.05	(0.13)		0.09	(0.14)		-0.07	(0.33)		1.70	(1.96)		1.70	(1.96)	
Wave 7 X 1 disability	-0.11	(0.06)		-0.05	(0.06)		-0.25	(0.09)	***	-0.12	(0.14)		-0.14	(0.13)		-0.08	(0.17)		-0.21	(0.22)		1.70	(1.96)	*	1.70	(1.96)	
Wave 7 X 2 disabilities	-0.11	(0.08)		-0.02	(0.08)		-0.29	(0.09)	***	-0.16	(0.16)		0.05	(0.16)		-0.15	(0.25)		-0.50	(0.32)		1.70	(1.96)		1.70	(1.96)	
Wave 7 X 3+ disabilities	0.24	(0.08)	**	0.35	(0.09)	***	-0.17	(0.13)		0.38	(0.22)		0.20	(0.19)		0.24	(0.20)		0.27	(0.34)		1.70	(1.96)		1.70	(1.96)	*

Notes. Total N ranged from 94616 to 95612

*** = p<.001; ** = p<.01; * = p<.05

Replicate weights were used in all analyses.

All models adjust for age.

© 2025 Parks MJ et al. *JAMA Network Open*.

eMethods. Methods for supplemental analyses in eTable 2.

Supplemental analyses consisted of weighted logistic regression models that used tobacco use measures as outcomes and tested whether there were interactions of time (wave) and number of disabilities for each tobacco use outcome. These interactions are formal tests for whether changes in tobacco use over time differed according to the number of disabilities (i.e., testing whether disparities remained the same or changed over time). Each model adjusted for age and included an interaction term that tested whether change across time depended on the number of disabilities. T-tests were used to test whether individual coefficients in the interaction term were statistically significant, and a design-adjusted joint Wald test was used to determine if the overall interaction was statistically significant.

Full model results are reported in eTable2. These models report individual coefficients for each interaction term. Design-adjusted, joint Wald tests showed that the overall interaction between time (wave) and number of disabilities were statistically significant for any tobacco use (F-score=5.64; p-value=<0.001), smokeless tobacco use (F-score=5.07; p-value=<0.001), e-product use (F-score=2.94; p-value=0.011), and cigarette use (F-score=5.05; p-value=<0.001). Even though there was an individual coefficient that was statistically significant for hookah use, the joint test was not statistically significant (F-score=2.08; p-value=0.063).

© 2025 Parks MJ et al. *JAMA Network Open*.
