# Supplementary material for: Tobacco and E-Product Use by US Adults With Disabilities
Source: JAMA Netw Open. 2025 Feb 18;8(2):e2460471. doi: 10.1001/jamanetworkopen.2024.60471 (PMC11836758; doi:10.1001/jamanetworkopen.2024.60471)
Supplement: Supplement 2. — Data Sharing Statement [file jamanetwopen-e2460471-s002.pdf]

Data Sharing Statement

Parks. Tobacco and E-Product Use by US Adults With Disabilities. *JAMA Netw Open*. Published February 18, 2025. doi:10.1001/jamanetworkopen.2024.60471

Data
Data available: Yes
Data types: Deidentified participant data
How to access data: All publicly available PATH data are available for download from ICPSR at the University of Michigan. https://www.icpsr.umich.edu/web/NAHDAP/series/606
When available: With publication

Supporting Documents
Document types: None

Additional Information
Who can access the data: Anyone who requests it.
Types of analyses: None.
Mechanisms of data availability: Publicly available PATH data are always available.
